# Camera footage and identification testimony undermine the availability of exculpatory alibi evidence

**DOI:** 10.1371/journal.pone.0289376

**Published:** 2023-10-26

**Authors:** Melanie Sauerland, Alana C. Krix, Katerina Georgiadou, Joke Humblet, Nick J. Broers, Anna Sagana

**Affiliations:** 1 Department of Clinical Psychological Science, Maastricht University, Maastricht, The Netherlands; 2 Department of Methodology and Statistics, Maastricht University, Maastricht, The Netherlands; University of Foggia: Universita degli Studi di Foggia, ITALY

## Abstract

The present field experiment investigated how alibi witnesses react when confronted with camera footage or identification testimony that incriminates an innocent suspect. Under the pretext of a problem-solving study, pairs of participants (*N* = 109) and confederates worked on an individual task with a dividing wall obstructing their view of each other. When the mobile phone of the experimenter was missing from an adjacent room at the end of the session, all participants confirmed that the confederate had not left the room. After several days, participants returned to the lab for a second session. They were asked to confirm their corroboration, orally and in writing, after learning that the confederate either had been identified from a photograph or was present on camera footage. A control group received no evidence. In this second session, written (but not oral) alibi corroboration was weaker in the incriminating evidence conditions (47%) than the no-evidence condition (81%), as hypothesized. Unexpectedly, corroboration was equally strong in the camera and identification evidence conditions. As expected, alibi corroboration was stronger in session 1 than in session 2 for both camera (89% and 31–46%) and identification evidence conditions (86% and 31–49%). The current findings provide first evidence that camera footage and eyewitness identification testimony can bear on the availability of exculpatory alibi evidence in court and emphasize the need to document incidents of evidence contamination.

## Introduction

Wrongful convictions can originate from flawed incriminating evidence and the absence of exculpatory evidence. Providing an alibi to someone is one such powerful piece of exculpatory evidence that can be lost because of context information. More specifically, although irrelevant for the task at hand, context information has the power to bias the evaluations of (forensic) evidence. For instance, consider a fingerprint or handwriting expert who becomes aware of a suspect’s confession. Although the confession itself should be irrelevant to the expert’s evaluation, knowledge of it increases the likelihood of interpreting non-matching fingerprints [1] or handwriting samples [2] as a match. This effect is known as *contextual bias*. However, how context information can shape a witness’s perception and decision-making in providing a stranger with an alibi is poorly understood. Here, we study how identification evidence and camera footage evidence can affect a witness’ willingness to provide an alibi to a stranger.

### Biasing effects of context information

A growing body of literature shows the significant impact of context information on the decision-making of forensic experts. Numerous studies have demonstrated that forensic evaluators, such as fingerprint and DNA analyst, odontologists and radiologists, can be influenced by extraneous context information [3–6]. For example, a fingerprint evaluation can be affected by knowledge of the result of a DNA analysis [6] or by an extraneous context that suggests that the prints are a non-match [7]. Similarly, irrelevant context information can bias odontology radiograph’s matching decisions [3] and forensic pathologists’ cause of death assessments [4]. Together, these studies show that context information has strong and consistent biasing effects on the experts’ evaluation of forensic evidence.

Another line of research suggests that context information may not only affect evaluations of evidence but also the evidence itself. For example, when eyewitnesses received information that the suspect confessed, they often changed their lineup identification decision to align with the confession [8], even if the perpetrator was not included in the lineup [9]. Similarly, when witnesses learned about the confession of an innocent suspect, they retracted their corroborating testimony in support of the suspect’s alibi [10]. These findings suggest that context information can compromise the independence of different pieces of evidence and even interfere with the availability of corroborating alibi testimony.

Contextual bias may also explain why exculpatory alibi statements are disregarded in real cases. The DNA Exonerations database (convictingtheinnocent.com) lists 156 cases of innocent convictions in which the defense presented an alibi as innocence defense. Whereas we cannot infer from the available case descriptions why these alibis were not believed, we do know that in John Kogut’s case, for instance, alibi witnesses withdrew their support when the police told them that the suspect had confessed [11]. Likewise, in the case of Barry Laughman, the police sent home two alibi witnesses who testified that they had seen the victim alive after the confessed murder allegedly occurred. It is possible that both Kogut’s and Laughman’s confessions corrupted the interpretation of subsequent information gathered in their cases and the believability of the alibis (cf. [11]).

Inspired by similar anecdotal data from real cases, Marion et al. [10] investigated the effect of context information on the availability of an exonerating alibi statement in the lab. After working with a confederate, participants learnt that money had been stolen from the adjacent office. A partition between the confederate and the participant, and the fact that the door of the laboratory was left ajar, introduced a certain ambiguity, simulating a situation where two people attend a social event together, but don’t stick together for the entire duration. Yet, upon hearing about the theft, 92% of the participants corroborated the confederate’s alibi stating that they had both been in the lab the whole time. When informed that the confederate had confessed to the theft, the rate of alibi corroboration among the initial corroborators dropped to 45%, compared to 95% in the condition where the confederate denied involvement. Alibi corroboration declined further to 20% when the experimenter led participants to believe that their support of the alibi might suggest that the participant had made a deal with the confederate. Interestingly, the corrupting effect of a confession on alibi corroboration seems to be equally strong in both strangers to and friends of the suspect [12]. These findings illustrate one possible pathway for the corruption of exculpatory evidence in proven cases of wrongful convictions such as those described above. This experiments shows how confessions can affect alibis. However, we know little about how other evidence affects alibi corroboration.

### The current study

The current study constitutes a conceptual replication of Marion et al. [10], extending this work to two other types of evidence: the suspect’s identification from a photograph and camera footage showing the suspect in the room where the theft took place. We selected these two types of evidence because they vary in the extent to which they are prone to subjective interpretation or what is commonly referred to as *elasticity*. More specifically, in the context of evidence, elasticity refers to the varying levels of uncertainty or the extent to which subjective interpretation can be applied to the evidence [13]. Camera footage is associated with low elasticity because video evidence is perceived to be an objective reflection of the events [14, 15]. Conversely, identifications are likely associated with high elasticity due to the many factors that can introduce error [16].

In this field experiment, participants who initially provided an alibi for a confederate returned to the lab after a delay of several days. We then informed participants either that another lab user had identified the confederate from a photograph or that the confederate had been seen on camera footage in the lab where the theft occurred. A control group received no evidence. We expected the context information, that is, the information about the identification or camera evidence, to create bias, such that alibi corroboration in session 2 would be stronger in the no-evidence group than in the groups that received incriminating information (hypothesis 1a). According to the concept of evidence elasticity, this effect should be stronger in the camera evidence condition (low elasticity) than the identification testimony condition (high elasticity; hypothesis 1b). Furthermore, both identification and camera evidence (but not the no-evidence condition) should decrease the strength of alibi corroboration, compared to initial corroboration provided in session 1 (hypothesis 2a) and this effect should be stronger in the camera footage condition than the identification testimony condition (hypothesis 2b). Finally, we expected participants in the no-evidence condition to believe more strongly that the confederate did not leave the room (hypothesis 3a) and to believe less strongly that the confederate was guilty of the theft (hypothesis 3b) than participants in the two evidence conditions. Table 1 provides an overview of the hypotheses.

**Table 1:** Overview of hypotheses.

Hypothesis 1a	Alibi corroboration in session 2 is stronger in the no-evidence group than in the groups that received incriminating information.
Hypothesis 1b	This effect is stronger in the camera footage condition (low elasticity) than the identification testimony condition (high elasticity).
Hypothesis 2a	Identification testimony and camera footage decrease the strength of alibi corroboration in session 2, compared to initial corroboration provided in session 1.
Hypothesis 2b	This effect should be stronger in the camera footage condition than in the identification testimony condition.
Hypothesis 3a	Participants in the no-evidence condition believe more strongly that the confederate did not leave the room than participants in the groups that received incriminating information.
Hypothesis 3b	Participants in the no-evidence condition believe less strongly that the confederate was guilty of the theft than participants in the groups that received incriminating information.

## Materials and method

### Participants and data exclusions

We aimed at a sample size of 37 participants per condition, based on a priori power analysis using G*Power [17, 18] for one-way ANOVA with three groups with power = .80, α = .05. In lack of studies to draw from to estimate the expected effect, we chose a moderate effect size of *f* = .30, under the assumption that a smaller effect size would not be meaningful. We pre-registered our hypotheses, criteria for data exclusion and inclusion, and analyses prior to data analyses on the open science framework (https://doi.org/10.17605/OSF.IO/QRHEK). We continued data collection until we reached the predetermined number of valid participants.

Participants were excluded if they did not return for session 2 (*n* = 7), did not corroborate the alibi in session 1 (*n* = 9), did not follow instructions (e.g., detached themselves from monitor; *n* = 3), or personally knew the confederate or experimenter (*n* = 2), if there was a technical failure (e.g., no recording for session 2 and no immediate protocol of statement; *n* = 4) or participants did not believe cover story (*n* = 69). We tested whether participants believed the cover story using two criteria: 1) did participants believe the phone was in fact stolen or did they think the theft was setup?; 2) were participants doubtful about the authenticity of the camera footage and identification testimony? If participants expressed doubts, experimenters prompted them to see if their doubts had emerged *during* the study or only now that they were asked these questions. We excluded participants who believed the theft to be a setup and those whose doubts about the authenticity were present *during* the study, that is, while dealing with the incident report form. We excluded another 63 participants from the analyses due to experimenter error (e.g., failure to explicitly accuse confederate, failure to mention phone was stolen). These exclusions were almost exclusively associated with one particular experimenter, despite elaborate training. The large number of errors transpired during the first round of transcript analyses. As pre-registered, we analyzed this group of participants separately. The general pattern of results held: oral corroboration, *p <* .001, Cohen’s *g* = 0.47, and written corroboration, *p* = .001, Cohen’s *g* = 0.39, were weaker in session 2 than session 1. In session 2, oral corroboration was weaker than written corroboration, *p* = .001, Cohen’s *g* = 0.44.

The final sample included 109 participants (19 men, 89 women, 1 non-identified; *M*_age_ = 20.5 years, *SD*_age_ = 2.1, *Mdn* = 20 years; age range 17–29 years), who were mostly students at the Faculties of Psychology and Neuroscience (79.8%) and Health Sciences (11.0%). Participants received course credit or a €7.50 gift voucher as reimbursement. The study was approved by the Ethics Review Committee Psychology and Neuroscience at Maastricht University (approval code ECP-159_06_12_2015). All participants provided written consent.

### Pilot study for selection of evidence differing in elasticity

To select types of evidence and to establish differences in elasticity, we conducted a pilot survey with *N* = 22 participants (14 women; *M*_age_ = 18.3 years, *SD*_age_ = 1.4, *Mdn* = 19 years; age range 18–23 years). Participants answered questions about nine different types of evidence including lineups, showups, DNA, fingerprints, camera footage, forensic ballistics, handwriting, microscopic hair, and bite mark analysis. Participants indicated how reliable they considered each type of evidence on a scale from 0 (*not at all reliable*) to 10 (*extremely reliable*). Next, they sorted the nine types of evidence in order from most (*1*) to least reliable (*9*) and indicated for each type of evidence how likely it was that it would be used in case of murder, violent assault, rape, robbery, theft, vandalism, or fraud. Participants also indicated who was qualified to collect the different types of evidence (*forensic specialists*, *police officers*, *anyone with the means to do so* or *other*). Here we report only those figures relevant for the current study. Full data are available here: https://doi.org/10.34894/XFSVPP.

Participants considered showup evidence the least reliable (*M* = 3.82, *SD* = 2.15, 95% CI [2.86, 4.77]) and DNA (*M* = 8.23, *SD* = 1.93, 95% CI [7.37, 9.08]), camera footage (*M* = 7.86, *SD* = 2.01, 95% CI [6.94, 8.77]), and fingerprint analysis (*M* = 7.50, *SD* = 1.79, 95% CI [6.71, 8.29]) the most reliable, *t*s(21/20) ≥ 8.55, *p*s < .001, *d*s ≥ 1.87, for the three comparisons with showup. Participants’ sorting order confirmed this finding (we report reversed scores for ease of interpretation): again, showups scored lowest (*M* = 2.14, *SD* = 1.06, 95% CI [1.66, 2.63]), whereas DNA (*M* = 8.71, *SD* = 0.64, 95% CI [8.42, 9.01]), fingerprint (*M* = 7.10, *SD* = 1.14, 95% CI [6.58, 7.61]), and camera footage analysis (*M* = 6.33, *SD* = 1.91, 95% CI [5.47, 7.20]) scored highest, *t*s(20) ≥ 9.20, *p*s < .001, *d*s ≥ 2.00, for the three comparisons with showup. A majority of participants thought that these methods would be used in case of a theft (showup: *M* = .58, *SD* = .51, 95% CI [0.33, 0.82]; DNA: *M* = .59, *SD* = .51, 95% CI [0.33, 0.85], fingerprints: *M* = .94, *SD* = .25, 95% CI [0.80, 1.07], camera footage analysis: *M* = 1.00, *SD* = .00, 95% CI [1.00, 1.00]).

Based on these findings, we initially selected the identification from a single photograph (i.e., resembling showup) as more elastic type of evidence and fingerprint analysis as less elastic type of evidence. We did not consider DNA because it would be difficult to convince participants that a DNA test had actually been conducted. However, following pilot testing, it became clear that participants would not believe that we actually conducted fingerprint analyses, either. We therefore used camera footage as less elastic type of evidence. This choice is supported by research in the elasticity of evidence that shows that camera evidence is considered less elastic than identification testimony [13, 19], Experiment 1.

### Design

We used a mixed 3 (type of evidence: no evidence vs. camera footage vs. identification testimony) x 2 (time of measurement: session 1 vs. session 2) design. Type of evidence was a between-subjects factor and time of measurement a within- subjects factor. The sample sizes of the three evidence conditions were *n* = 37, 36, and 36. The strength of alibi corroboration in session 1 (oral) and session 2 (oral and written) and the differences between them (session 1 vs. session 2; oral vs. written) served as dependent measures. We also measured participants’ beliefs that the confederate did not leave the room and that she was guilty of the theft.

### Procedure

**Fig 1:**
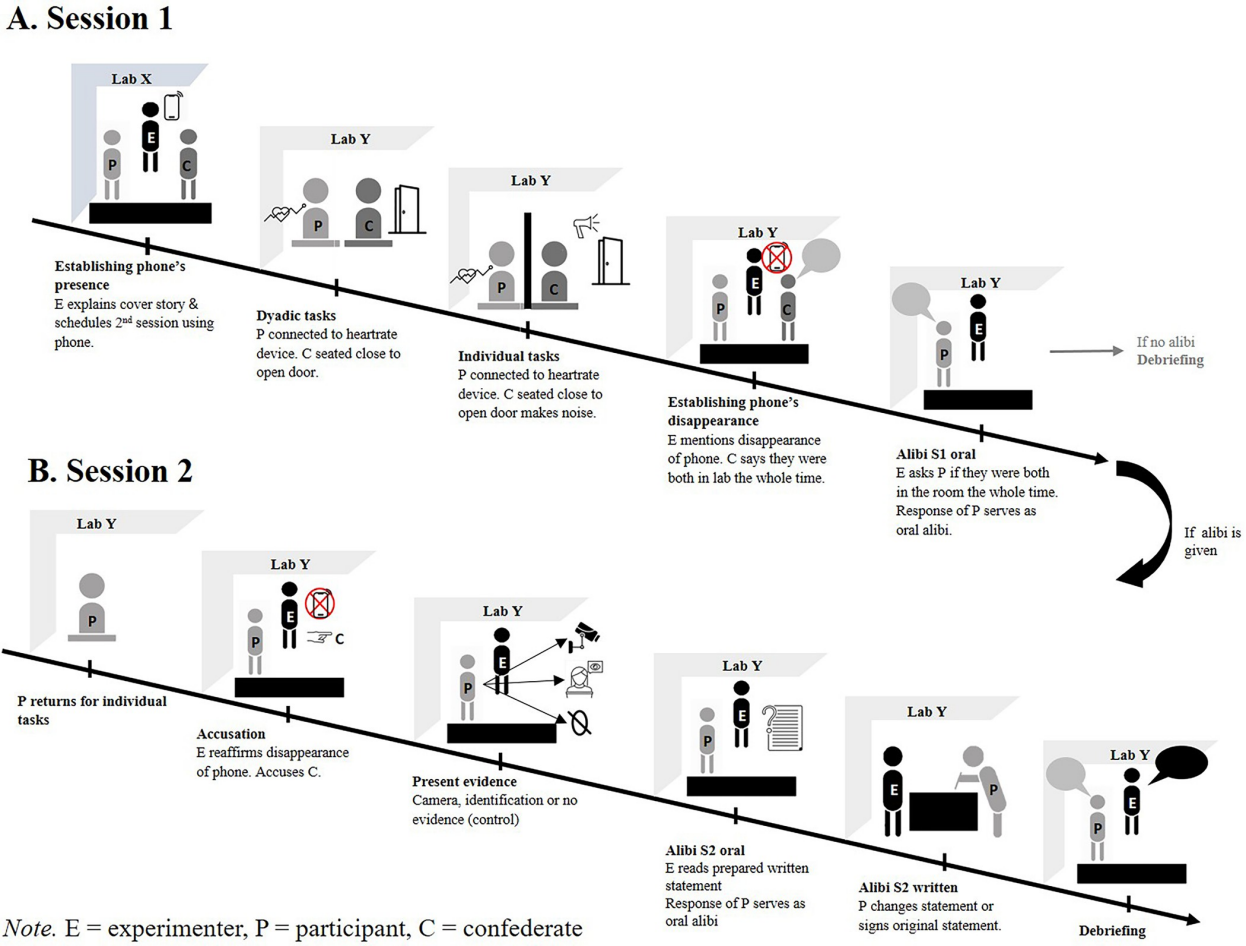
Overview of experimental procedure.

The procedure was based on Marion et al. [10]. Fig 1 shows an overview.

### Session 1

The experimenter greeted the participant and the confederate and provided them with consent and demographic data forms. As a cover story, the experimenter informed them that the study tested how different sleeping patterns related to performance on individual and dyadic problem-solving tasks. The experimenter used her smartphone to schedule session 2 with each of them and left it on top of a desk. After setting a time and date, the experimenter led both the participant and the confederate to a different lab (lab Y), leaving her phone on the table in the first lab (lab X).

#### Dyadic problems

When entering lab Y, the confederate made sure to sit in the chair close to the door, so that the participant would sit on the spot that was equipped with a heart-rate monitor. The experimenter then mentioned a “pilot study” they were currently running “on the physiology of problem-solving” and asked the participant if it was okay to attach them to the monitor using a finger clip. The confederate would also agree, but the experimenter would ask the participant if they minded, because they were closer to the device.

The experimenter then provided the pair with the dyadic problem-solving tasks. The experimenter then exited the lab but left the door open, mentioning that it could get quite hot in the lab. The confederates who were blind to the experimental conditions, acted politely, but not overly friendly or likeable towards the participant (cf. [10]). The confederate would give only brief responses and would not give much input on solving the dyadic problems to avoid that a relationship that could affect the likelihood of alibi corroboration would form between the two.

#### Individual problems

After seven minutes, the experimenter returned, handed out the individual problems and told the pair to work on their own. She also put a dividing wall between the two, so they could not see each other and so that the participant could not see the door. When exiting the experimenter left the door open. The confederate made some noises throughout, such as clicking a pen and moving in her chair to create the impression that leaving the room would have been difficult, but not impossible for the confederate.

#### First alibi corroboration

Another seven minutes later, the experimenter returned and, to support our cover story, provided two sleep questionnaires [20, 21] and some problem-solving questionnaires. The experimenter returned after about five minutes and casually mentioned that her phone was missing, that she was worried that someone might have taken it, and whether the two of them had heard or seen anything or anyone in the lab. The confederate answered that they had both been in the lab. Before disconnecting the participant from the monitor, the experimenter reminded them of the appointment for the second session and excused the confederate. When the confederate had left, the experimenter asked the participant if they were sure that they had both been in the lab the whole time. Upon participants’ confirmation, the experimenter downplayed the incident by saying that she had probably misplaced the phone, and the session ended. If the participants did not confirm, the experimenter debriefed them. The response of the participants was covertly audio recorded. Session 1 lasted 25–35 min.

### Session 2

Session 2 took place about 3–6 days later as an individual session (i.e., without the confederate). To maintain the cover story, the experimenter provided the participant with a new set of individual problem-solving tasks and three questions about the quality of their sleep in the previous night. When the experimenter returned, she announced completion of the experiment. She then mentioned that she had not found her phone after session 1, that she suspected it had been stolen, and that she had to fill in an incident report form. The experimenter explained that she knew that the participant could not have taken the phone because they had been attached to the monitor the whole time, but that she suspected the confederate.

#### Presentation of evidence

In the identification condition, the experimenter added that another researcher who used the lab across had seen someone enter lab X and had identified the confederate as being that person from a photograph fetched from Facebook. In the camera condition, the experimenter stated that she had asked security to look at the camera footage from lab X and that she had seen the confederate enter and exit the lab. However, because the camera was directed at the door she could not see whether the confederate took the phone.

#### Second alibi corroboration

The experimenter presented the incident report form, read the form with the participant and asked the participant to sign the form as a witness. The experimenter stressed that the confederate had denied stealing the phone. The experimenter then orally enquired whether the participant could still confirm that both participants had been in lab Y during the whole session. If the participant confirmed, the experimenter asked him or her to sign. If the participant did not confirm, the experimenter asked if they wanted to change the statement. Participants would then amend the statement at their own discretion and sign it. Finally, participants estimated how strongly they believed that the other participant did not leave the room and that the other participant was guilty of the theft on a scale from 1 (*not at all*) to 10 (*definitely*). In the end, the experimenter asked participants not to share information with others about the study, debriefed the participant, and provided the consent form for the use of their audio recordings. Session 2 took about 20 min.

### Materials

#### Problem-solving tasks

The dyadic problem-solving task consisted of seven logical reasoning problems (e.g., "How can you add eight 8s to get the number 1000 [using only addition])? Three different versions were used through the course of the experiment to introduce novelty for the confederates from time to time. The individual tasks in session 1 consisted of 20 anagrams. The individual tasks in session 2 consisted of 20 word association problems.

#### Problem-solving questionnaire

In line with the cover story, participants answered questions regarding the problems they had solved earlier (e.g., *The dyadic/individual tasks were difficult*; *I enjoyed working on the dyadic/individual tasks*).

*Confederate likability*. The problem-solving questionnaire also contained nine items to measure the likability of the confederate. These items were based on the Reysen Likability Scale [22]. We adapted item 6 to better match the situation in the experiment (*I would not mind working together with the other participant in the future* rather than *I would like this person as a coworker*) and dropped item 3 and 7. The remaining items were *The other participant was friendly/ likable/ agreeable/ approachable*; *I would ask the other participant for advice (in general)*; *I would like to be friends with the other participant*; *The other participant was similar to me; and The other participant was knowledgeable*. Participants rated these items on a scale from 1 (*strongly disagree*) to 5 (*strongly agree*). We combined the responses into one confederate likability scale by summing up the answers (Cronbach’s α = .88). The score can range between 9 and 45.

#### Incident report form

This two-page bogus form with the university header, contained two sections and was similar to the form used by Marion et al. ([10]; see Appendix for an example). Section A consisted of a hand-written statement of the theft. The description mentioned that the participant had been attached to the heart-rate device for the entire duration of the study and that the confederate had denied committing the theft. In the identification condition, the description additionally mentioned that another researcher had seen someone enter lab X and that this person had identified the confederate from a photograph. In the camera condition, the description additionally mentioned that the experimenter had seen the confederate enter lab X on the recording from the camera installed in that lab. The experimenter’s name was printed underneath and the experimenter had already signed and dated the form. In section B, there was space for three witness statements. In one field, the experimenter had already written down the witness statement that confirmed that the confederate had been in lab Y during the entire duration of the experiment. In the identification condition, there was a second signed witness statement from the person who had recognized the confederate from a photograph.

#### Believability of cover story and evidence

Participants answered questions that prompted whether they had believed the cover story: *What do you think is the purpose of this study*? and *Was there anything you found strange about this study*? When submitting those questions, participants received another set of questions that were slightly more suggestive than the preceding open items: *Did you think the other participant acted strangely (when you worked with her)*?; *Did you find it odd that you were the only one hooked up to the heart monitor*?; *Did you believe the phone was stolen*? Participants in the identification and camera conditions additionally received questions about the believability of the evidence.

### Coding of alibi corroboration and data analyses

Participants’ oral corroboration statements in session 1 were coded as being *strong* (5; e.g., “yes, we were both here”), *modest* (4; attenuations addition, e.g. “I think so”), or *weak* (3; e.g., “I don’t know/I wasn’t paying attention, but I think she was here”). Oral and written statements provided in session 2 were coded analogously, with the additional categories: *no corroboration* (2; e.g., “I don’t know/I wasn’t paying attention”) and *weak incrimination* (1; e.g., “It could be she left the room indeed”). Two coders independently coded the strength of corroboration for 173 randomly selected corroboration statements from session 1 and 2 (i.e., 53% of the statements). Disagreements were resolved in discussion. Cohen’s κ across all those statements was κ = .80, *p* < .001, and κ = .75 for oral statements in session 1, κ = .82 for oral statements in session 2, and κ = .74 for written statements in session 2, *p*s < .001.

Inspection of the data revealed extreme skewness. In session 1, the vast majority of participants (87.9%) scored 5 on the corroboration scale, 8.4% scored 4, and 3.7% scored 3. Session 2 revealed greater dispersion in the scores, with a smaller majority scoring 5 (oral corroboration: 39.4%; written corroboration; 58.7%) and a small minority scoring 1 (oral corroboration: 0.9%; written corroboration; 3.7%). The overall distribution remained very skewed (scoring 2: oral: 26.6%; written: 15.6%, scoring 3: oral: 11.0%; written: 12.8%; scoring 4: oral: 22.0%; written: 9.2%). Therefore, deviating from the pre-registration but similar to Marion et al. ([10]; who also used a 3-point scale, i.e., no, weak, and strong corroboration), we collapsed corroboration levels 3 and 4 (*corroboration but some doubts*) and corroboration levels 1 and 2 (*no corroboration*) for comparing alibi corroboration in session 2 (hypotheses 1a/b). Because the new scale is ordinal rather than continuous, we opted for multinomial regression analyses, because this method is more suited for our data than the pre-registered analyses of variance (ANOVA).

Because we did not include participants who did not corroborate the alibi in session 1 (i.e., observations for categories 1 and 2), a further concatenation of categories was required to enable a comparison of alibi corroboration between sessions 1 and 2 (i.e., to test hypotheses 2a/b). To this end, we dichotomized corroboration to 1 (*strong* corroboration) vs. 0 (absence of strong corroboration). Although this deviates from the pre-registration, this is again analogous to Marion et al. [10] who also employed a dichotomous corroboration for their analyses of session 2. We compared the distributions for each evidence condition using McNemar’s tests. Cohen’s *g* served as a measure of effect size. Conventionally, effects < 0.20, between 0.20 and 0.25, and > 0.25 can be interpreted as small, moderate, and large, respectively [23]. To compare participants’ beliefs that the confederate did not leave the room and that the confederate was guilty of the theft (hypotheses 3a/b), we conducted ANOVAs, as pre-registered.

### Results

On average, the delay between session 1 and 2 was 4.61 days (*SD* = 1.39, 95% CI [4.35, 4.88]). Delay did not differ significantly as a function of type of evidence condition, *F*(2, 105) = 0.69, *p* = .503, *η*_*p*_^*2*^ = .013. The average confederate likability score was 34.48 (*SD* = 5.77, 95% CI [33.38, 35.58]) and *M* = 35.04 (*SD* = 4.77, 95% CI [34.12, 35.96]) after removing three outliers (scores ≤ 19). Confederate likability did not differ significantly across type of evidence conditions, *F*(2, 102) = 0.73, *p* = .486, *η*_*p*_^*2*^ = .014. Neither delay nor confederate likability correlated statistically significantly with any of the three corroboration measures, |*r*|s ≤ .19, *p*s ≥ .055. We conducted the subsequent analyses with and without the three confederate likability outliers. This did not affect the pattern of results. In the following, we therefore report analyses on the full sample. Five participants said they believed the phone was missing rather than stolen (i.e., believed the cover story, but thought the experimenter had misplaced the phone). The patterns of results were analogous, with one exception, as specified. We report analyses for the full sample.

### Effect of presented evidence on alibi corroboration in session 2 (hypothesis 1a and 1b)

Table 2 shows strength of alibi corroboration as a function of type of evidence for both sessions. Hypothesis 1a predicted alibi corroboration in session 2 to be stronger in the no-evidence group than the camera and identification conditions. Hypothesis 1b predicted this effect to be stronger in the camera footage than the identification testimony condition. To test the effect of type of evidence (none vs. camera vs. identification) on alibi corroboration (none vs. some doubts vs. strong) in session 2, we computed two multinomial regression analyses, one for written corroboration and one for oral corroboration. Indicating differences in *written* corroboration across evidence conditions, the overall model was significant, Σ^*2*^(4) = 14.37, *p* = .006. Table 3 shows inferential statistics for post-hoc comparisons. In support of hypothesis 1a, these comparisons showed that participants in the no-evidence condition were more likely (81.1%) to provide strong corroboration than participants in the two other conditions (47.2%). Contrary to hypothesis 1b, corroboration in the camera vs. the identification conditions did not differ. For *oral* corroboration, the overall model was statistically non-significant, Σ^*2*^(4) = 7.15, *p* = .128, lending no support for hypothesis 1a or 1b (no-evidence: 56.8%; camera and identification: 30.6%).

**Table 2:** Frequency of different levels of alibi corroboration (proportion in brackets) in session 1 and 2 as a function of type of evidence.

	Strong corroboration *n* (%)	Some doubts *n* (%)	No corroboration *n* (%)
Session 1^1^	94 (87.85)	13 (12.15)	n/a
Session 2 –written			
No evidence	30 (81.08)	5 (13.51)	2 (5.41)
Identification	17 (47.22)	11 (30.56)	8 (22.22)
Camera	17 (47.22)	8 (22.22)	11 (30.56)
Across conditions	64 (58.72)	24 (22.02)	21 (19.27)
Session 2 –oral			
No evidence	21 (56.76)	8 (21.62)	8 (21.62)
Identification	11 (30.56)	14 (38.89)	11 (30.56)
Camera	11 (30.56)	14 (38.89)	11 (30.56)
Across conditions	43 (39.45)	36 (33.03)	30 (27.52)

^1^ Two recordings were missing; therefore the sample size for session 1 is 107.

**Table 3:** Parameter estimates (logodds) for multinomial regression of the effect of evidence condition (none vs. camera vs. identification) on written corroboration (none vs. some vs. weak) in session 2.

	*B*	Std. Error	Wald Σ^2^	*p*
No corroboration (= reference category) vs. some corroboration				
No evidence^1^ vs. identification	-0.598	.96	0.39	.532
No evidence^1^ vs. camera	-1.235	.96	1.67	.197
Camera vs. identification^1^	-0.637	.66	0.94	.332
No corroboration (= reference category) vs. strong corroboration				
No evidence^1^ vs. identification	-1.954	.85	5.33	.021*
No evidence^1^ vs. camera	-2.273	.83	7.56	.006**
Camera vs. identification^1^	-0.318	.58	0.30	.581
Some corroboration (= reference category) vs. strong corroboration				
No evidence^1^ vs. identification	-1.356	.62	4.80	.028*^2^
No evidence^1^ vs. camera	-1.038	.64	2.58	.108
Camera vs. identification^1^	0.318	.58	0.30	.581

* *p* < .05:

** *p* < .01

^1^ reference category

^2^ *p* = .069 (*B* = -1.156) when *n* = 5 participants who thought the phone was *missing* rather than *stolen* were excluded

### Effect of elasticity on development of alibi corroboration between session 1 and 2 (hypothesis 2a and 2b)

Hypothesis 2a predicted weaker alibi corroboration in session 2 vs. 1 when participants received incriminating evidence. Hypothesis 2b predicted this effect to be stronger in the camera than the identification evidence condition. For *written* corroboration, the findings supported hypotheses 2a: for the camera, *p* < .001, Cohen’s *g* = 0.50, and the identification conditions, *p* = .002, Cohen’s *g* = 0.38, the proportion of participants who provided a strong alibi in session 2 was smaller than in session 1 (camera: 88.6% vs. 45.7%; identification: 85.7% vs. 48.6%). The sizes of these effects were large. On the other hand, there was no statistically significant difference in the rate of strong alibis in session 1 vs. 2 in the no-evidence condition, *p* = .508, Cohen’s *g* = 0.17 (89.1% vs. 81.1%). Contrary to hypothesis 2b, the effects for the camera and the identification conditions did not differ significantly from another, Σ^*2*^(2, *N* = 70) = 2.11, *p* = .605, Cramer’s *V* = .17.

For *oral* statements, the proportion of participants who provided strong alibi corroboration in session 2 was smaller than in session 1 for all evidence conditions, with large effect sizes throughout (no-evidence: *p* = .004, Cohen’s *g* = 0.38, 89.2% vs. 56.8%, camera: *p* < .001, Cohen’s *g* = 0.50, 88.6% vs. 31.4%, identification: *p* < .001, Cohen’s *g* = 0.45, 85.7% vs. 31.4%). Again, the size of the effects in the camera and the identification conditions did not differ significantly, Σ^*2*^(2, *N* = 70) = 1.03, *p* > .999, Cramer’s *V* = .122. These findings lend support to hypotheses 2a, but not 2b.

### Effect of presented evidence on participants’ belief of absence and guilt in session 2 (hypotheses 3a and 3b)

Hypothesis 3a predicted that participants’ belief that the confederate did not leave the room would be stronger in the no-evidence than the camera and identification conditions. Hypothesis 3b predicted that participants’ belief that the confederate was guilty of the theft would be weaker in the no-evidence vs. the camera and identification conditions. Across conditions, participants generally believed that the confederate did not leave the room (*M* = 7.67, *SD* = 2.07, 95% CI [7.28, 8.07]) and the belief that the confederate was guilty of the theft was low (*M* = 3.35, *SD* = 2.23, 95% CI [2.92, 3.77]). These ratings did not differ as a function of type of evidence, *F*s(2, 106) ≤ 0.77, *ps ≥* .466, *η*_*p*_^*2*^s ≤ .014, lending no support to hypotheses 3a and 3b.

## Discussion

In a field experiment, we tested the hypothesis that contextual information can bear on the availability of exculpatory alibi evidence. Participants who provided an alibi for a confederate received context information in the form of either camera footage or eyewitness identification testimony. We expected this context information to create bias such that alibi corroboration after hearing the evidence would be weaker compared to a no-evidence condition (hypothesis 1a) and that corroboration would decrease, compared to alibi witnesses’ initial corroboration statement (hypothesis 2a).

### Effects of incriminating evidence on alibi corroboration (hypotheses 1a and 2a)

Our results support our hypotheses for written alibi corroboration. Written alibi corroboration statements were significantly weaker in the incriminating evidence conditions (47.2%) than the no-evidence condition (81.1%) in session 2 (hypothesis 1a). Furthermore, compared to the initial corroboration statement in session 1, strong alibi corroboration occurred significantly less often in written alibi statements when participants received incriminating evidence (camera: 88% in session 1 vs. 42% in session 2; identification: 85% vs. 46%), whereas written alibi corroboration remained equal in strength in the no-evidence group (89% vs. 75%, hypothesis 2a).

Oral alibi corroboration in session 2 did not differ as a function of evidence condition, contrary to hypothesis 1a. This was because, in line with hypothesis 2a, strong oral alibi corroboration occurred less often in session 2 (30–53%) than session 1 (85–89%) across all evidence conditions and not only in the incrimination conditions. These findings extend earlier reports that demonstrated the damaging effect of context information on the availability of exculpatory alibi evidence [10, 12], the quality of eyewitness testimony [8, 9], and the quality of evidence evaluations (e.g., [24–26]).

### Effects of formal status on alibi corroboration

Another interesting finding concerns the effect of formal status on corroboration. We coded what participants *said* during the conversation with the experimenter (oral corroboration) and the statement participants eventually *signed* (written corroboration) in session 2 separately. Across all evidence conditions, strong *oral* alibi corroboration occurred less often than strong *written* alibi corroboration. That means, participants displayed skepticism about the innocence of the confederate in speaking with the experimenter, but when it came down to the formal reporting and signing of a written statement that matched their doubts, participants were not ready to do so. Possibly, participants empathized with the experimenter and conformed with her suggestion in the direct conversation with her. However, they may have considered the costs and consequences of withdrawing their support for the alibi or making an accusation in *written* as too high, also because they had confirmed the alibi with some confidence just days earlier. Participants’ hesitation for changing their written statement matches the finding that participants generally believed that the participant had *not* left the room and had *not* committed the theft, and–contrary to hypotheses 3a and 3b –these beliefs were not moderated by type of presented evidence.

### Effects of evidence elasticity on alibi corroboration (hypotheses 1b and 2b)

We also tested the hypothesis that the elasticity associated with different types of evidence moderates the effect of context information [13, 19]. Based on the idea that the elasticity of camera footage is weaker than the elasticity of identification testimony, we expected alibi corroboration in session 2 to be stronger in the identification than the camera condition (hypothesis 1b) and we expected camera footage to have a more damaging effect on alibi corroboration than identification testimony (hypothesis 2b). Our findings did not support these hypotheses. Possibly, elasticity of our camera footage condition was stronger than intended, because it did not show the actual theft–a decision we made in order to maintain a certain level of ambiguity in the evidence presented. Future studies could identify the conditions that determine differences in the perception and therefore elasticity of identification and camera footage evidence.

### Decision making and constraint satisfaction

The present findings could be seen through the lens of constraint-satisfaction [27, 28] and, more specifically, the *coherence effect* [29]. At a neuronal and computational level, constraint-satisfaction posits that mentally represented variables relevant for a task constrain each other in a way that drives the representation toward a state of global coherence. Positively linked variables (e.g., two agreeing pieces of evidence: a belief and evidence that matches the belief) excite one another, whereas negatively linked variables (e.g., two opposing pieces of evidence: a belief and evidence that contrast the belief) inhibit each other until the system settles at a point of maximum coherence. Thus, the main characteristic of a system operating by constraint satisfaction is that it allows a decision to be reached by consensus of evidence. In the context of decision-making, this happens by gradually shifting one’s perception of the task towards a state of coherence with one of the decision alternatives. The direction of the shift depends on the weight of the factors that are relevant to the decision at hand. These factors include the strength of the facts, but also background knowledge and the belief system of the decision-maker. This is a bidirectional decision-making process in which the decision-maker not only interprets the factors but also uses emerging interpretations to reappraise the weight of the factors. The result is progressive polarization: the decision maker devalues factors that discord with the emerging decision but augments factors that support the emerging decision.

In legal decision-making, probative evidence (e.g., camera footage) tends to assimilate other uncertain, ambiguous, and conflicting elements (e.g., one’s perception of the actions of another person) as to accord with the evidence [30]. In the present study, in the absence of context information (i.e., session 1), the belief that the participant remained in the lab sufficed to elicit a strong corroboration decision. The presentation of contradicting context information in session 2 introduced a decisional conflict. Participants likely resolved the conflict through constraint satisfaction: by gradually shifting their corroboration decision to be consistent with the emerging evidence that they presumably considered most probative. Further research might investigate the effect of coherence effects on alibi corroboration more directly by asking participants to rate the strength of each piece of evidence and the probability of guilt after the presentation of each (for a similar approach see [31, 32].

### Practical and policy implications

The current findings have important implications for our understanding of the genesis of wrongful convictions. Thus far, we knew that investigator and confirmation bias can corrupt evidence [24], resulting in biased evaluations of different pieces of evidence. Two recent studies showed that confession evidence has an impact not only on the *evaluation* of evidence, but also on the very *availability* of exculpatory evidence [10, 12]. The current findings show that an exculpatory alibi undermining effect is not limited to confession evidence.

The troubling issue with alibi evidence is that–once shared–context information can contaminate or weaken the memory of the alibi witness (much like new information can contaminate the memory of other eyewitnesses). Thus, an interview with an alibi witness cannot be revised and redone once the alibi witness received the new information. As a result, investigators and legal practitioners likely assign little or no exculpatory weight to the now uncorroborated alibi. This is likely what happened in the cases of Kogut and Laughman [11] and many other cases (convictingtheinnocent.com). Thus, the absence of (corroborated) alibi evidence may well result from a combination of the effects of context information on alibi witnesses and investigators.

We can also derive implications for legal procedure and policy from the current study. The corruptive effects of context information on alibi evidence could be mitigated, or avoided altogether, by using blind procedures and recording interactions with alibi witnesses [24]. Additionally, documentation is crucial to identify whether contamination happened and of what nature it was. In cases of intelligence interviewing, where the strategic presentation of evidence is advised and cannot be avoided (e.g., [33, 34]), documentation should detail the moment and the type of information that was presented to the witness. If a witness withdraws the alibi, investigators should document and explicitly communicate the initial corroboration statement to jurors and judges. Legal decision-makers can then use this information to determine whether corruption of evidence was an issue and weight the presented evidence accordingly. It is important to realize, though, that jurors and other decision-makers may not be able to fully understand the effects of irrelevant context information on the presented evidence [35].

### Limitations and future directions

Type of evidence had a differential effect on the comparison between strength of corroboration in session 1 vs. 2 only when looking at written, but not oral corroboration in session 2. This could be considered a limitation of the alibi-undermining effect of context information. One the other hand, a written alibi statement has formal status and requires a higher level of commitment from alibi witnesses and is therefore most relevant when considering the effect of context information on the availability of alibi corroboration in a real case. Furthermore, alibi corroboration in session 2 differed as a function of type of evidence, regardless of corroboration mode. An interesting line of research worth investigating concerns the question in how far contradictive evidence can undermine the availability of exculpatory evidence other than a corroborated alibi. For example, are there some types of evidence that generally trump other types and is this mostly a question of timing or a combination of both?

Another critique refers to the external validity of the setting. Indeed, the theft of a mobile phone constitutes a relatively minor crime that would unlikely reach the prosecution level. Note, however, that larceny and property theft are the most common crimes in the Netherlands [36] and the US [37], emphasizing the relevance of our scenario for real life. Similarly, we did not involve police officers or other figures of authority that would normally be involved in an investigative process. On the other hand, the number of participants who did not believe the theft was real was substantial, possibly leading to a selection bias. Both limitations are inherent in experimental research that aims to study actual *behavior* in a field experiment rather than *self-reports* of what participants *think* they might do in a certain situation (cf. [38]). We can speculate that the real effect of corrupting evidence on alibi corroboration might be somewhat smaller for more severe crimes but somewhat larger when figures of authority conduct the interviews with the witness. We can also speculate that the high number of participants not believing the cover study is owed to the setting, namely testing mostly psychology students who know that they are participating in a study and may have heard about staged events. Future studies might therefore choose a different setting where participants are not aware a priori that they are participating in a study–although there are ethical issues involved. Despite all those considerations, the current data do provide a first estimate of people’s actual behavior when they receive alibi corrupting information.

Finally, a word on generality is appropriate. The current results derive from a western European, academic, mostly Caucasian, mostly female, young sample. Wrongful convictions, on the other hand, often happen to people with lower socio-economic status and people of color. It is possible that the demographics of our sample had an effect on alibi corroboration. Although speculative, there could be a bias to provide a stronger alibi to women than men or to same-race suspects vs. different-race suspects. These are interesting questions that might be addressed in future studies.

To conclude, the current study demonstrates that camera footage and identification testimony have the power to undermine the availability and weaken the strength of exculpatory alibi evidence. This is troubling because there are currently no procedures in place to prevent wrongful convictions by loss or devaluation of exculpatory evidence. Avoidance and documentation of possible evidence contamination incidents should be a top priority for those concerned with criminal justice.
